# Evaluation of Fast and Sensitive Proteome Profiling of FF and FFPE Kidney Patient Tissues

**DOI:** 10.3390/molecules27031137

**Published:** 2022-02-08

**Authors:** Irena Dapic, Naomi Uwugiaren, Jesper Kers, Yassene Mohammed, David R. Goodlett, Garry Corthals

**Affiliations:** 1 International Centre for Cancer Vaccine Science, University of Gdansk, 80-309 Gdansk, Poland; naomiuwugiaren@gmail.com (N.U.); goodlett@uvic.ca (D.R.G.); 2 Van’t Hoff Institute for Molecular Sciences, University of Amsterdam, 1012 WP Amsterdam, The Netherlands; j.kers@amsterdamumc.nl; 3 Department of Pathology, Amsterdam Infection & Immunity Institute (AI & II), Amsterdam Cardiovascular Sciences (ACS), Amsterdam UMC, University of Amsterdam, 1012 WP Amsterdam, The Netherlands; 4 Department of Pathology, Leiden University Medical Center, 9600 Leiden, The Netherlands; 5 Genome BC Proteomics Centre, University of Victoria, Victoria, BC V8Z 5N3, Canada; Yassene@proteincentre.com; 6 Center for Proteomics and Metabolomics, Leiden University Medical Center, 9600 Leiden, The Netherlands; 7 Department of Biochemistry and Microbiology, University of Victoria, Victoria, BC V8Z 5N3, Canada; 8 Department of Pathology, Amsterdam University Medical Centre, University of Amsterdam, 1012 WP Amsterdam, The Netherlands; 9 Maastricht MultiModal Molecular Imaging Institute, Maastricht University, 6229 ER Maastricht, The Netherlands

**Keywords:** FF, FFPE, mass spectrometry, kidney, MS-compatible detergents

## Abstract

The application of proteomics to fresh frozen (FF) and formalin-fixed paraffin-embedded (FFPE) human tissues is an important development spurred on by requests from stakeholder groups in clinical fields. One objective is to complement current diagnostic methods with new specific molecular information. An important goal is to achieve adequate and consistent protein recovery across and within large-scale studies. Here, we describe development of several protocols incorporating mass spectrometry compatible detergents, including Rapigest, PPS, and ProteaseMax. Methods were applied on 4 and 15 μm thick FF tissues, and 4 μm thick FFPE tissues. We evaluated sensitivity and repeatability of the methods and found that the protocol containing Rapigest enabled detection of 630 proteins from FF tissue of 1 mm^2^ and 15 μm thick, whereas 498 and 297 proteins were detected with the protocols containing ProteaseMax and PPS, respectively. Surprisingly, PPS-containing buffer showed good extraction of the proteins from 4 μm thick FFPE tissue with the average of 270 protein identifications (1 mm^2^), similar to the results on 4 μm thick FF. Moreover, we found that temperature increases during incubation with urea on 4 μm thick FF tissue revealed a decrease in the number of identified proteins and increase in the number of the carbamylated peptides.

## 1. Introduction

In the emerging field of tissue proteomics, biopsies and sections from organs are being used to convert solid tissues to molecular and digital information. After removal from the patients, tissues are stored as either formalin-fixed paraffin-embedded (FFPE) or fresh frozen (FF) tissues, often in optimal cutting temperature (OCT) medium. Formalin fixation and paraffin-embedding has been a standard method used for preservation of the tissues and recently FFPE tissues have also been used to reveal cancer biomarkers [1,2,3], analysis of glycoproteomes with MS in cancer [4], and genetics [5]. However, due to chemical reactions that take place following formaldehyde induced cross-linking and over time during preservation, intra- and intermolecular interactions within the tissue and protein matrices occur. Chemical modifications of proteins add difficulty to achieving excellent protein recovery from FFPE tissues, as the scope of number of modifications are poorly described. However, progress and new knowledge has grown in recent years as a number of studies have detailed the potential impact of the FFPE preservation procedure [6,7,8] with respect to the discoverable proteome.

Already as a part of routine procedures, paraffin from FFPE tissues is removed by use of xylene and water [9] and tissues are subsequently incubated at an elevated temperature (95 °C) to maximize protein retrieval [10,11]. Additionally, extraction buffers have been supplemented with detergents to enhance protein solubility and recovery. While SDS is one of the most commonly used detergents for protein extraction in protein biochemistry [12], its incompatibility with ESI-MS and MALDI-MS has limited its use. As an alternative, several new detergents have emerged, such as Rapigest [13,14], PPS [15], or ProteaseMax [16], which are compatible with LC/ESI-MS procedures. Here, we investigated their effectiveness in tissue analysis. 

The motive for use of detergent is to release efficiently and comprehensively (all) proteins from their cellular matrix into the aqueous phase ready for subsequent MS analysis. Here, yield and efficiency are important parameters. Yield, in particular, is important as FF and FFPE tissues are limited by size and the sheer amount of tissue available for molecular studies. Therefore, sample loss, due to partial protein extraction, directly has an effect on the detection and quantitation of proteins. 

There are also other methods emerging that aim to improve the detection limits in proteomics. Techniques, such as micro- and nano-liter scale sample preparation methods [17,18,19] which with laser capture microdissection (LCM) can be developed into spatial proteome mapping systems exist [20]. Several other technologies have included integrated proteome analysis devices such as “iPAD” for on-line digestion of 100 cells [21], immobilized enzyme reactors (IMERs) [22,23,24,25], and, in a blast from the past, capillary zone electrophoresis (CZE) [26] has recently shown to improve efficiency of sample preparation.

Nevertheless, in-solution sample preparation is currently the most widely used approach for analysis of human tissues, probably due to its ease of use. The variety of tissue types and ongoing challenges to establish a ‘universal’ method for protein retrieval from FFPE tissues requires constant attempts and community evaluation to move towards defining method(s) that allow for a generality of analysis of tissues [27]. Subsequent adaption by colleagues in pathology labs may then follow. 

**Figure 1:**
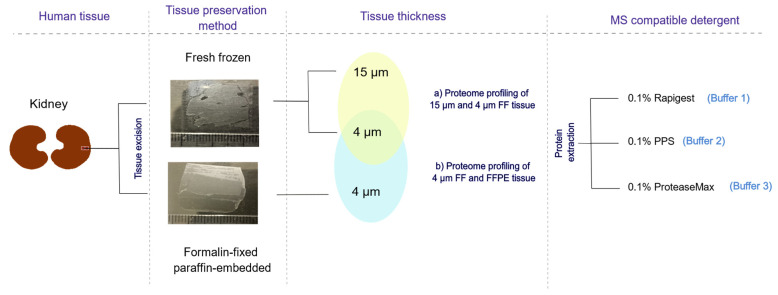
Scheme of the study of protein extraction from the FF and FFPE tissues with MS-compatible buffers. Proteins were analyzed using 4 μm and 15 μm thick FF tissue to examine effect of the tissue thickness on the protein identification with several different methods. Further, identified proteins were compared between 4 μm thick FF and FFPE tissues to evaluate appropriateness of the minute amounts of the FFPE tissues to be used with the MS-compatible protocols.

In this study, we analyzed biopsy-size tissue sections, which are 4 μm thick and are routinely used by pathologists world-wide. Our ambition was to develop fast and robust sample preparation for 4 μm thick human kidney biopsy tissues, ideally FFPE. We used both FF and FFPE tissues to investigate what difference could be found in protein detection between tissue storage types (Figure 1). Results showed that methods were sensitive enough to detect proteins from only several mm^2^ of 4 μm thick tissue. To investigate sensitivity and reproducibility related to the amount of tissue, 4 μm thick FF tissue was compared to 15 μm thickness of the same tissue. Further, we investigated the proteome profile of paired 4 µm FF and FFPE tissues to determine if 4 µm FFPE tissues were suitable for diagnostic and classification purposes. Similar physicochemical properties in terms of distribution of molecular weight, GRAVY score, and pI values were detected for proteins in both tissue types, whereas most of the proteins (265 ± 44) were detected in 4 µm thick when PPS was used.

## 2. Materials and Methods

### 2.1. Materials and Reagents

NH_4_HCO_3_ was obtained by Fluka (Zwijndrecht, The Netherlands), acetonitrile (ACN, LC-MS grade), formic acid (FA), and water (ULC/MS) were purchased from Biosolve (Valkenswaard, The Netherlands). Iodoacetamide (IAA, ≥99%), dithiothreitol (DTT, ≥99%), acetone, urea, trifluoroacetic acid (TFA, ≥99%), and trypsin (European Pharmacopoeia reference standard) were delivered by Sigma Aldrich (Zwijndrecht, The Netherlands). PPS Silent Surfactant™ was obtained from Expedeon, ProteaseMax from Promega and Rapigest SF from Waters.

### 2.2. Kidney Tissue Samples

FFPE and FF tissues of the kidney were provided by Amsterdam University Medical Centers in Amsterdam anonymously. In short, small, equal-sized excisions were prepared from a transplant nephrectomy specimen. FF tissues were snap-frozen in optimal cutting temperature medium (OCT) and subsequently stored at −80 °C until analysis. FFPE tissues were fixated overnight in 4% buffered formalin, the next day embedded in paraffin and subsequently stored at room temperature until analysis. Tissue slides were generated with a standard clinical pathology lab microtome at 4 μm and 15 μm micrometer thickness. Tissue area of the excised material on slides was measured using ImageJ software (version 1.49). All experiments were performed in triplicate.

### 2.3. Tissue Preparation for LC-MS

FFPE tissues were first deparaffinized by incubating the glass slide containing the excised tissue in xylene for 2 min to remove the paraffine. Next, the tissue was incubated for 2 min in a gradient of EtOH with the following order: 100% EtOH, 85% EtOH, 70% EtOH, and finally deionized water.

The FF and FFPE tissues were cut out with the scalpel from microdissection and scraped off into the Eppendorf tube containing extraction buffer. Proteins from the tissues were extracted with the following buffers: 1) 0.1% Rapigest, 30% ACN, and 8 M urea in 100 mM NH_4_HCO_3_ (Buffer 1); 2) 0.1% PPS, 30% ACN, and 8 M urea in 100 mM NH_4_HCO_3_ (Buffer 2); and 3) 0.1% ProteaseMax, 30% ACN, and 8 M urea in 100 mM NH_4_HCO_3_ (Buffer 3) using the following procedures. Samples were first incubated in 50 μL of the buffers with no urea addition for 90 min at 95 °C. After the first extraction, samples were cooled down to room temperature, 150 μL of 8 M urea was added and samples were further incubated for 30 min at 37 °C. Subsequently, 2.5 μL DTT (700 mM) was added to each sample and incubated for 30 min at 37 °C. Further, 9.2 μL IAA (700 mM) was added to the samples and incubated for 30 min at 37 °C. Finally, samples were diluted with 120 μL of 1M NH_4_HCO_3_ and 880 μL of H_2_O. To each sample, a total of 70 ng of trypsin was added and proteins were incubated for 17 h at 37 °C. After digestion, samples were purified on an SPE cartridge and stored at −20 °C until instrumental analysis.

### 2.4. NanoLC/ESI-MS/MS

For the analysis of the tissue samples an Eksigent Ekspert nanoLC 425 system (Sciex) coupled to the nanoelectrospray interface (nanoESI) of a TripleTOF 5600+ was used. Peptides were loaded onto an Eksigent trap column (nano-LC trap set, ChromXP C18, 120 Å, 350 μm, 0.5 mm) and further desalted with 3% ACN and 0.1% FA at rate 2 μL/min. Subsequently, peptides were submitted to an in-house packed analytical column (Magic C18 resin, 100 Å pore size, 5 μm particles, 75 μm i.d., 10 cm column length) at 300 nL/min. Peptides were eluted with the mobile phase consisted of 5−40% B for 45 min, 40−95% B for 5 min, 95% B for 9 min, and finally 95–5% B in 1 min. Mobile phase A was composed of 0.1% formic acid (FA) in H_2_O, and mobile phase B of 0.1% FA in ACN. Detection of the peptides was performed in intensity-dependent acquisition (IDA) mode, and survey scans were acquired in 500 ms, with *m*/*z* from 400 to 1250 Da. In each duty cycle, 30 product ion scans were collected for 100 ms in the *m*/*z* range from 200 to 1800 Da, if exceeding 100 counts per seconds and for the charge state 2+ to 4+. During acquisition, dynamic exclusion was used for half of the peak width and rolling collision energy was used. 

Samples were analyzed on LC-MS so that for 15 μm thick tissue amount corresponding to 1 mm^2^ of the tissues was analyzed (corresponding to a small clinical biopsy) and for 4 μm thick amount corresponding to 2.5 mm^2^ of the tissue was analyzed.

### 2.5. Data Processing

Raw data files (.wiff) were converted into .mgf format using MS Data Converter (Beta 1.3, https://ab-sciex-ms-data-converter.software.informer.com/1.3b/, accessed on 7 August 2021). Files were processed using SearchGUI (version 3.3.16, http://compomics.github.io/projects/searchgui, accessed on 7 August 2021) and searched with X!Tandem against the Uniprot database (downloaded September 5, 2019). Carbamidomethylation of cysteine residues was set as fixed modification and methionine oxidation was set as variable modification. Carbamylation of K residues and protein N-termini were set as variable modifications for carbamylation analysis, and for up to two missed cleavages were allowed for tryptic digestion. Results were analyzed using PeptideShaker [28] (version 1.16.42, http://compomics.github.io/projects/peptide-shaker, accessed on 7 August 2021), and proteins at 1% FDR were filtered. The protein and peptide identification lists were exported from PeptideShaker for each sample and the results of the different samples corresponding to replicates were aggregated according to the protein accession number or the peptide sequence. In the case of calculating the total number of proteins, the union of the protein IDs was used. Otherwise, the individual counts for each replicate were used and represented as measurement spread in the individual plots. Protein sequences and protein annotations concerning subcellular localization were retrieved from UniProtKB (http://www.uniprot.org, accessed on 7 August 2021), GRAVY scores were determined using the GRAVY calculator (http://www.gravy-calculator.de, accessed on 7 August 2021) and pI values were obtained using ExPASy (https://web.expasy.org/compute_pi/, accessed on 7 August 2021).

## 3. Results and Discussion

### 3.1. Evaluation of Protein Extraction with Different Tissue Thicknesses and Protocols

**Figure 2:**
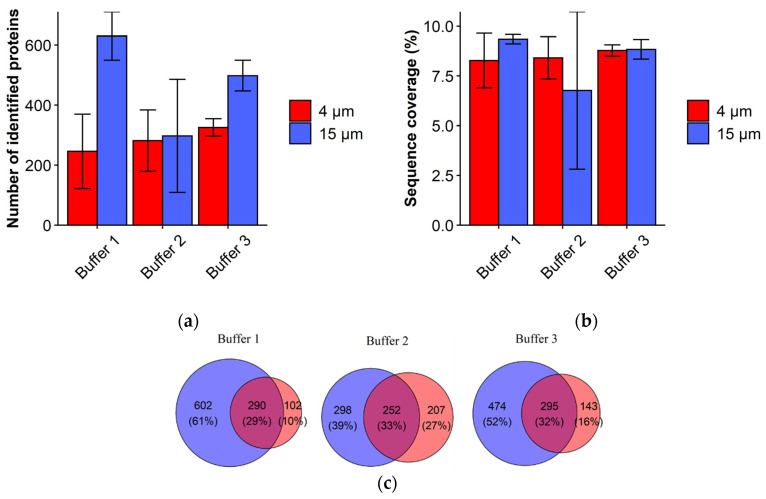
Qualitative and quantitative comparison of the proteins extracted from 4 and 15 µm thick FF tissue using Buffers 1 to 3. (**a**) Number of identified proteins; (**b**) Sequence coverage; (**c**) Venn diagrams illustrating the distribution of all identified proteins between 15 µm (blue) and 4 µm (red) thick FF tissue. Results are shown as mean ± SD.

Protein extraction and further detection from the complex samples are influenced by the composition of the extraction buffer and the digestion procedures. Use of the MS-compatible detergents in extraction buffers might improve unfolding and solubility of the proteins while avoiding detergent interference with MS detection. In our study, several protein extraction protocols have been applied to investigate protein extraction from 4 µm thick tissue and to compare it with the results from 15 µm thick tissue. Buffers used for protein extraction varied in composition of MS-compatible detergents (Rapigest, PPS, and ProteaseMax) as described in Methods and Materials section. As shown in Figure 2a, Buffers 1 and 3 yielded the highest number of proteins identified for 15 µm tissue (1 mm^2^), while fewer proteins were identified in corresponding samples of 4 µm tissue (2.5 mm^2^). However, similar sequence coverage results were obtained for 4 and 15 µm tissue suggesting that buffers have similar efficiencies in solubilizing proteins for both tissue thicknesses (Figure 2a). Correlation of the NSAF values of the identified proteins between the biological replicates showed good correlation ranging from 0.76 to 0.9 for Pearson coefficients (Figure S1, see Supplementary Materials) indicating good reproducibility of the methods.

Qualitative method comparison was measured in terms of protein overlap (Figure 2c). The number of uniquely identified proteins was highest for 15 µm tissue using Buffer 1 and 3, while the overlap between 4 and 15 µm tissue was similar using Buffers 1, 2, and 3.

Overall, these results demonstrate that protein extraction from 4 µm tissue is possible and that protein extraction with different protocols was efficient enough for detection of high numbers of proteins from 4 µm tissue compared to 15 µm tissue.

### 3.2. Evaluation of Extraction Buffer Efficiency and Reproducibility in FF and Archival FFPE Tissues

**Figure 3:**
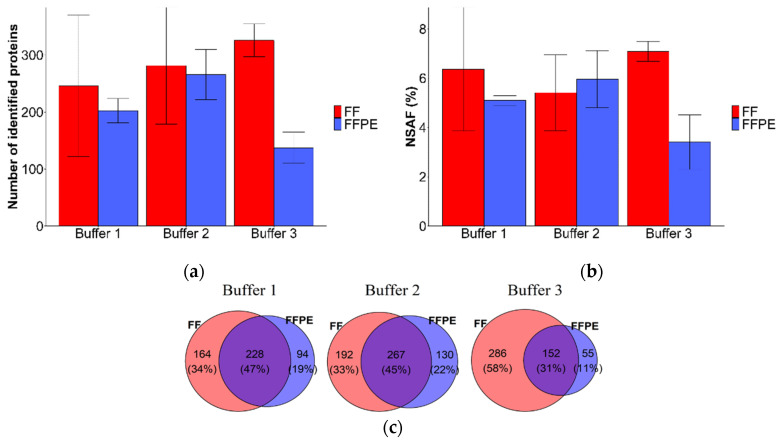
Qualitative and quantitative comparison of 4 µm thick FF and FFPE tissue. (**a**) Number of identified proteins for 4 µm thick FF and FFPE kidney tissue with Buffers 1 to 3. (**b**) Quantitative comparison of protein abundance by total NSAF values for 4 µm thick FF and FFPE tissue after extraction with Buffers 1 to 3. NSAF values were expressed as a percentage of all proteins in all samples of all buffers. (**c**) Venn diagrams illustrating distribution of identified proteins between 4 µm thick FF (red) and FFPE (blue) tissue samples for Buffers 1 to 3. Results are shown as mean ± SD.

To compare efficiency in protein extraction for different tissue types Buffers 1, 2, and 3 were applied to 4 µm thick FF and FFPE tissues. Results showed that higher protein identifications and NSAF values were observed for FF tissues using Buffer 3 (Figure 3a,b), while the number of protein identifications and NSAF values were similar for FF and FFPE tissues using Buffer 1 and 2. Venn diagrams in Figure 3c illustrate the overlap of protein identifications among 4 µm FF and FFPE tissue samples for the three buffers. The highest overlapping protein identifications between 4 µm thick FF and FFPE tissues were obtained for Buffer 1 and Buffer 2 (47% and 45%, respectively), while the number of uniquely identified proteins was highest for FF samples using Buffer 3 (58%).

Overall, the results of 4 µm FF and FFPE tissues are comparable from which we can conclude that FFPE tissues could be utilized when FF tissues are not available. Buffer 2 was selected for further analysis as this buffer showed the highest efficiency for protein extraction for 4 µm FFPE tissues and gave comparable results for different tissue thicknesses and types. 

Qualitative and quantitative reproducibility of the methods were assessed between replicates of 4 µm thick FF and FFPE tissue (Figure S1, see Supplementary Materials) using Buffer 2. Qualitative reproducibility was evaluated in terms of protein identification overlap between biological replicates which was 16% and 25% for FF and FFPE tissue, respectively. Semi-quantitative reproducibility was determined by calculating the Pearson correlation coefficients between replicates and results showed good correlation with r ≥ 0.85 for both FF and FFPE tissue.

### 3.3. Comparison of Physicochemical Properties of Identified Proteins

**Figure 4:**
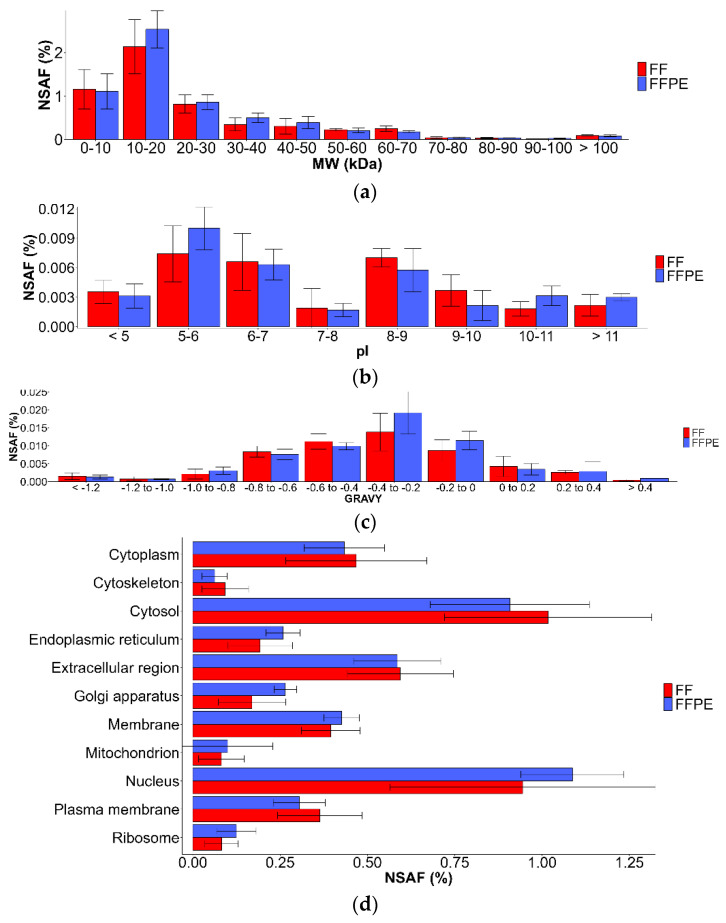

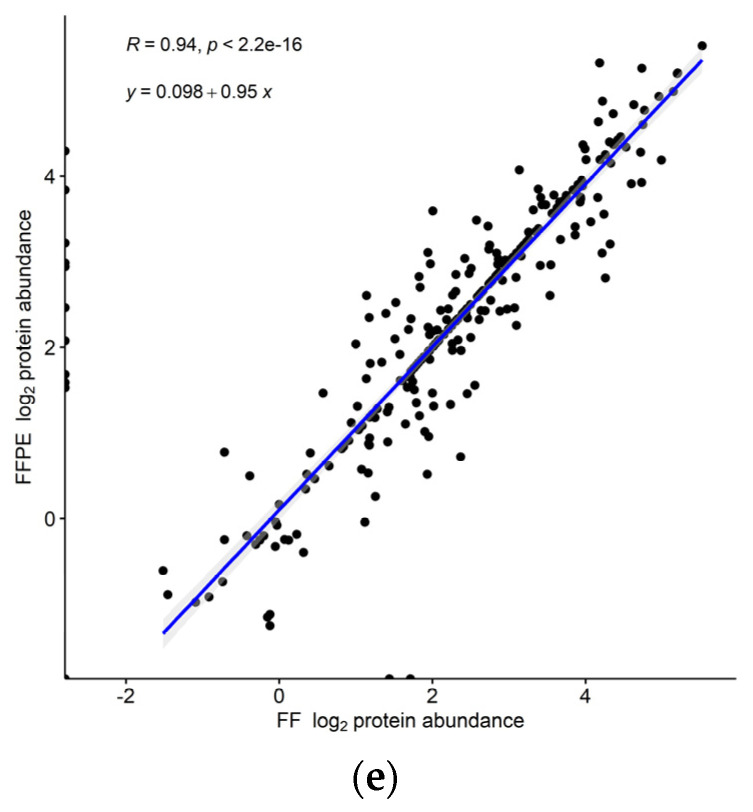
Comparison of NSAF values according to physicochemical properties of the identified proteins from 4 µm thick FF and FFPE tissue after the extraction with Buffer 2 according to: (**a**) *M*_W_, (**b**) pI, (**c**) GRAVY scores, and (**d**) cellular localization. NSAF values are expressed as a percentage of total proteins; results are shown as mean ± SD; (**e**) correlation of the protein abundance for proteins detected with Buffer 2 in 4 µm thick human kidney FF and FFPE tissues.

The physicochemical properties of the identified proteins extracted with Buffer 2 were evaluated in terms of molecular weight (*M*_W_) distribution, pI distribution, GRAVY scores, cellular localization, and K/R ratio. For both FF and FFPE tissues, the most abundant proteins are between 10 and 20 kDa mass range (Figure 4a). Proteins within the mass range 10–50 kDa had higher abundances in FFPE tissues compared to corresponding FF tissues. Further, as shown in Figure 4b, NSAF values were highest for proteins with pI values 5–6, followed by the proteins with a pI values of 6–7 and 8–9. Figure 4c shows the distribution of the identified proteins in terms of their GRAVY scores. The most abundant proteins were detected in the range −0.4 to −0.2 for both FF and FFPE tissue. FFPE samples contained a higher abundance of proteins in the range of −0.4 to 0 GRAVY score. The semi-quantitative distribution of the proteins was determined as their NSAF values according to their cellular localization (Figure 4d). Similar abundancies between the proteins were observed among FF and FFPE tissues, with proteins in the cytosol and nucleus having the highest abundance, while membrane proteins were slightly more abundant in FFPE samples which could be indication of good solubilization properties of Buffer 2. Cellular localization and NSAF values of the proteins identified in FF and FFPE tissues with Buffer 2 are shown in Supplementary Table S1. Moreover, further comparison showed good correlation of abundance of the proteins between FF and FFPE tissues (Figure 4e) which could indicate appropriateness of 4 μm FFPE tissues in this study.

**Figure 5:**
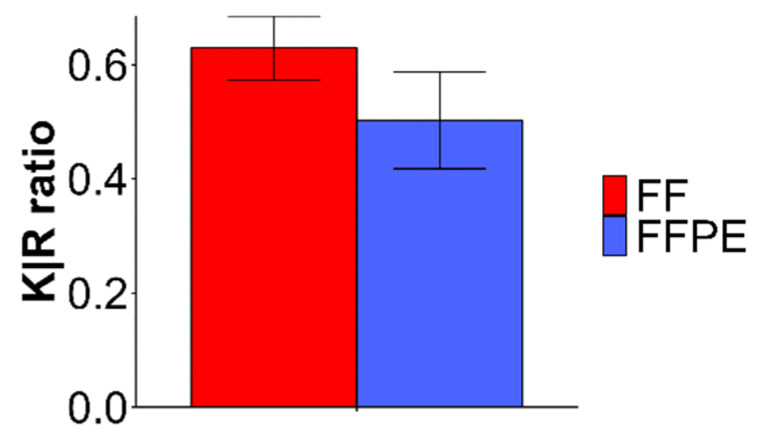
Ratio of C-terminal lysine-containing peptides versus C-terminal arginine-containing peptides (K/R) after samples processing with Buffer 2.

Further, results for the ratio of C-terminal lysine-containing peptides versus C-terminal arginine-containing peptides (K/R) are shown in Figure 5. Results show that K/R was decreased for FFPE compared to FF tissue which is in agreement with the previously published literature [29,30,31,32]. Underrepresentation of K residues due to the reaction of side chains with formaldehyde confirm that lysine side chains are more involved in cross-linking reactions with formaldehyde, to date mostly studied on tissues sections in the range of 8–60 μm [29,30,31], while previously for 3 μm tissues micro sized heat-induced antigen retrieval chamber was used [32].

### 3.4. Effect of Temperature on Carbamylation during Urea-Incubation

**Figure 6:**
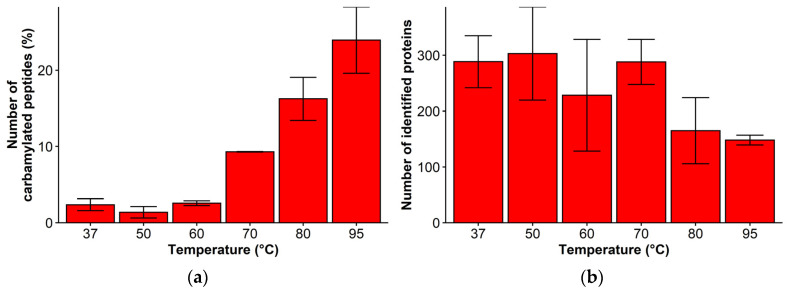
Influence of temperature on peptide and protein carbamylation for 4 µm FF kidney tissue during incubation in 30% ACN/8 M urea/100 mM NH_4_HCO_3_ at 37, 50, 60, 70, 80, and 95 °C. (**a**) Increase in the percentage of carbamylated peptides; and (**b**) Decrease in number of identified proteins during incubation with urea. Results are shown as the mean ± SD.

Incubation with urea-containing buffers might lead to peptide carbamylation and hindered protein digestion due to side reactions with isocyanic acid, which is enhanced at elevated temperatures [33,34,35]. Modifications of the proteins might induce incomplete protein digestion and alternate chromatographic analysis of the peptides. The influence of an elevated temperature during incubation was tested by incubating 4 µm FF tissue samples at increasing temperatures with the buffer containing 30% ACN/8 M urea in 100 mM ammonium bicarbonate whereas, previously, ammonium bicarbonate has been shown as carbamylation inhibitor [33]. Increasing incubation temperatures of the FF tissues in the buffer increased the percentage of carbamylated peptides which consequently led to a decrease in the number of identified proteins as shown in Figure 6. These results indicate that in the case of antigen retrieval of the FFPE tissues at elevated temperatures, urea should be supplemented into the buffer in subsequent steps after decreasing the temperature of the mixture to 37 °C.

## 4. Conclusions

Detection of the proteins from the human tissues is important for patient care and diagnostics. FFPE tissues are the most common method for storing patient material and it is essential to find a reliable and reproducible protocol(s) for protein retrieval from this type of material. We specifically developed the protocol so it could be directly used on biopsy samples stored in pathology, even at surfaces below 2.5 mm^2^. The rationale was to enable incorporation of the workflow without the need to take an extra biopsy (which is the case for most RNA-based techniques). 

In this study, we examined protein retrieval and compared results for 15 µm thick FF kidney tissues, and applied developed protocols to 4 μm thick FF and FFPE kidney tissue sections. We observed that the most proteins were identified in 15 μm FF tissues with a buffer containing Rapigest (630 ± 80), followed by ProteaseMax (498 ± 51), and finally PPS (297 ± 188). However, from 4 µm FFPE tissues, most proteins were recovered (265 ± 44) with a buffer containing PPS, suggesting this buffer to be appropriate for screening of the proteins in small tissue amounts. Proteomics of thin sections of FFPE tissues has already been employed on different tissue types [13,36,37] to investigate protocols with the goal to optimize them to obtain patient data. While there are existing methods as pressure cycle technologies which omit long digestion times [37], in-solution digestion due to its low cost and simplicity has still been most widely used. Additionally, in our study, proteins from 4 µm FF and FFPE tissues showed similarity in physicochemical properties indicating appropriateness of the used in-solution protocol for protein extraction from FFPE tissues.

Our results on kidney FFPE tissues showed underrepresentation of lysine (K) residues, probably due to a reaction of the lysine sidechains with formaldehyde in FFPE tissues, which is in agreement with previously published work [31,37]. Significance of the experimental optimization of the protocols which use urea in protein extraction is also demonstrated in previous reports whereas maximum yield was observed after samples were incubated at elevated temperature (95 °C), and urea was added after decreasing the temperature of the sample solution to 60 °C [38]. However, in the case of the lysate of SK-MEL cells, rat spleen lysates, and pancreatic tumor lysates, the best performance was observed when digestion was performed with urea at room temperature [35]. Here, we observed that urea at elevated temperature, which is necessary for recovery of proteins from FFPE tissues, results in an increase in the number of carbamylated peptides, while the number of the identified proteins decreased. This implies that urea should only be used later in the protocol with incubation at lower temperature.

## Data Availability

The mass spectrometry proteomics data have been deposited to the ProteomeXchange Consortium via the PRIDE partner repository with the dataset identifier PXD020793.

## Supplementary Materials

The Supporting Information contains qualitative and quantitative reproducibility of the results, physicochemical properties of proteins detected in all methods. Table S1. Peptides list and Proteins list. The mass spectrometry proteomics data have been deposited to the ProteomeXchange Consortium via the PRIDE [39] partner repository with the dataset identifier PXD020793. Figure S1. Qualitative and quantitative reproducibility of protein extraction from (a) 4 µm thick FF and FFPE kidney tissue, and (b) 15 µm thick FF kidney tissue using Buffers 1 to 3. Venn diagrams show the distribution of identified proteins among biological replicates and correlation of the log 10 NSAF values between replicates is shown using Pearson r coefficient. Figure S2. Quantitative analysis of physicochemical properties of the identified proteins from 4 µm thick FF and FFPE tissue using Buffers 1 to 3. Quantitative protein distribution in terms of number of protein identifications according to (a) MW, (b) pI, (c) cellular localization.

## Abbreviations

ACN	acetonitrile
CZE	capillary zone electrophoresis
DTT	dithiothreitol
EtOH	ethanol
FA	formic acid
FF	fresh frozen
FFPE	formalin-fixed paraffin embedded
GRAVY	grand average of hydropathy
IAA	iodoacetamide
IMER	immobilized enzyme reactors
iPAD	integrated proteome analysis devices
LCM	laser capture microdissection
NSAF	normalized spectral abundance factor
OCT	optimal cutting temperature
TFA	trifluoroacetic acid
